# Is There Still a Role for Twist Drill Craniostomy in Contemporary Management of Chronic Subdural Hematoma?

**DOI:** 10.3390/brainsci16050516

**Published:** 2026-05-12

**Authors:** Hussam Hamou, Hani Ridwan, Anna Mausberg, Roel Haeren, Hans Clusmann, Anke Hoellig, Michael Veldeman

**Affiliations:** 1 Department of Neurosurgery, RWTH Aachen University Hospital, 52074 Aachen, Germany; 2 Department of Diagnostic and Interventional Neuroradiology, RWTH Aachen University, 52074 Aachen, Germany; 3 Department of Neurosurgery, Maastricht University Medical Center, 6229 HX Maastricht, The Netherlands

**Keywords:** chronic subdural hematoma, twist drill craniostomy, burr hole craniotomy, recurrence, hematoma architecture, propensity score matching

## Abstract

**Background/Objectives**: Chronic subdural hematoma (cSDH) is an increasingly prevalent neurosurgical condition in the aging population. Burr hole craniotomy (BHC) with irrigation and postoperative drainage represents the evidence-based standard of care, yet recurrence rates remain substantial. Twist drill craniostomy (TDC), a minimally invasive bedside procedure performed under local anesthesia, offers theoretical advantages for frail patients but has been largely abandoned due to concerns regarding incomplete evacuation and recurrence. This study aimed to identify the predictors of a successful TDC outcome and to compare the recurrence rates between TDC and BHC. **Methods**: We performed a retrospective cohort study of consecutive patients undergoing surgical treatment for radiologically confirmed cSDH at RWTH Aachen University Hospital between 2015 and 2023. Hematoma morphology was classified using an extended CT-based architecture system and grouped into homogeneous, organized, sedimented, or subacute categories. The primary endpoint was recurrence requiring surgical reintervention. Multivariable logistic regression was used to identify independent predictors of recurrence among patients discharged after definitive TDC. Propensity score matching was performed to compare recurrence rates between TDC and BHC while adjusting for baseline demographic, clinical, and radiographic differences. **Results**: Among 178 patients initially treated with TDC, 56 (31.5%) were discharged without conversion to BHC. Late recurrence occurred in 26 of 56 patients (46.4%) treated definitively with TDC. In multivariable analysis, homogeneous hematoma architecture was the only independent predictor of recurrence (adjusted OR 4.48, 95% CI 1.10–22.07, *p* = 0.037). Propensity score matching yielded 48 well-balanced pairs of TDC and BHC patients. Recurrence rates remained significantly higher after TDC compared with BHC (42.6% vs. 17.0%, *p* = 0.012), as confirmed by conditional logistic regression (adjusted OR 3.20, 95% CI 1.17–8.73). **Conclusions**: Twist drill craniostomy may provide definitive treatment in carefully selected patients but is associated with substantially higher recurrence rates than burr hole craniotomy, particularly in homogeneous hematomas. Burr hole evacuation remains the preferred standard approach, while optimized drainage protocols and architecture-guided selection may define a limited role for TDC in high-risk patients.

## 1. Introduction

Chronic subdural hematoma (cSDH) is an increasingly common neurosurgical condition, particularly in the aging population [1,2]. The standard of care, as established by multiple randomized controlled trials, consists of burr hole craniotomy under general anesthesia with irrigation using body-temperature saline and postoperative drain placement [3,4,5]. Despite optimization of the surgical technique, recurrence rates remain substantial, with approximately 10 to 30% of patients requiring repeat surgery [6,7,8].

The demographic shift toward an older patient population presents a clinical challenge. Patients with cSDH frequently present with multiple comorbidities, increasing the perioperative risks associated with general anesthesia. Local anesthesia and conscious sedation can be used as an alternative approach in observational studies associated with shorter length of hospital stay and duration of surgery [9]. Conscious sedation carries its own limitations, including suboptimal head positioning, increased risk of pneumocephalus, and potential need for emergency intubation due to respiratory insufficiency. Furthermore, this elderly population is particularly vulnerable to postoperative delirium, a complication strongly associated with anesthetic exposure [10].

Twist drill craniostomy (TDC) represents an alternative treatment modality that can be performed at the bedside under local anesthesia. This procedure involves creating a small burr hole (3.5–4.5 mm) and allowing passive drainage of the hematoma through suction, irrigation, or gravity alone [11,12,13]. The procedure can be performed immediately without requiring anesthesiologist availability, the duration is short, it provides rapid relief of mass effect and associated symptoms, it avoids anesthesia-related risks, and it is highly cost-effective. As such, TDC is particularly of interest, as it may address some of the healthcare capacity challenges related to cSDH care.

Recent advances in understanding cSDH pathophysiology suggest that hematoma organization and internal architecture may reflect the degree of fibroproliferation occurring within the hematoma cavity, a process that can be conceptualized as the body’s intrinsic healing mechanism [14,15]. We have previously demonstrated that more organized hematomas, as classified by computed tomography (CT) imaging characteristics, are associated with lower recurrence rates following standard surgical treatment. This observation raises the question of whether there is a subset of patients with favorable hematoma characteristics in whom TDC would provide adequate treatment while avoiding the risks of general anesthesia.

The present study aims to identify the clinical and radiographic characteristics that predict a successful outcome following TDC. By phenotyping patients who achieve durable resolution without recurrence after this minimally invasive procedure, we seek to determine whether TDC may have a continued role in carefully selected patients, particularly those at high anesthetic risk with hematoma features suggestive of active spontaneous resolution.

## 2. Materials and Methods

### 2.1. Study Population and Design

This retrospective cohort study analyzed consecutive patients who underwent surgical treatment for chronic subdural hematoma (cSDH) at RWTH Aachen University Hospital between January 2015 and December 2023. The current investigation extends our previously reported institutional experience and uses overlapping patient cohorts with prior studies [16,17]. The specific emphasis of this investigation lies in identifying predictors of recurrence following TDC and comparing outcomes between TDC and BHC approaches. The institutional ethics review board provided approval (EK 20/399 and EK 25/331), and the retrospective design warranted a waiver of individual informed consent. Study registration was completed with the German Clinical Trials Register (DRKS00025280).

The inclusion criteria required radiologically confirmed cSDH on CT with subsequent surgical evacuation via either BHC or TDC. Patients were excluded if preoperative CT imaging was unavailable; if intracranial hypotension contributed to collection formation; if previous cranial operations or interventions were etiologically linked to the hematoma; or if non-iatrogenic coagulopathy (including hepatic dysfunction or hereditary bleeding disorders) was present.

### 2.2. Clinical Data Acquisition

Patient information was systematically retrieved from the institutional electronic medical record system. The extracted clinical variables encompassed demographic characteristics, traumatic injury history, presenting symptoms and neurological deficits, Glasgow Coma Scale (GCS) at admission, and baseline comorbid conditions. Preadmission pharmaceutical regimens were recorded with specific documentation of antiplatelet agents (single-agent and combination therapy) or oral anticoagulants (vitamin K antagonists and direct oral anticoagulants). Initial laboratory assessments included activated partial thromboplastin time, international normalized ratio, and platelet count.

### 2.3. Imaging Assessment

Preoperative CT scans underwent standardized radiological evaluation for several imaging parameters. Measurements included hematoma location (unilateral or bilateral), maximal transverse width, craniocaudal dimension, and volumetric quantification (Brainlab, Munich, Germany).

Hematoma internal organization was categorized using our institution’s previously published extended classification system comprising eight morphological subtypes: bridging, subacute, laminar, trabecular, hyperdense, isodense, hypodense, and sedimented [14]. Classification was performed independently by two investigators (HH and MV) who were masked to patient outcomes. For statistical analysis, the eight subtypes were collapsed into four broader categories: homogeneous (including hypodense, isodense, and hyperdense morphologies), organized (incorporating laminar, bridging, and trabecular patterns), sedimented, and subacute.

### 2.4. Treatment Protocol and Perioperative Care

Operative intervention was pursued for patients demonstrating neurological compromise (including motor deficits, ambulatory dysfunction, language disturbance, or epileptic seizures) or for neurologically intact patients showing radiographic mass effect (such as midline shift, ventricular effacement, or sulcal obliteration). Headache as an isolated symptom without objective neurological findings or imaging evidence of mass effect did not constitute a surgical indication.

The conventional surgical technique involved BHC with saline irrigation and insertion of one or two passive subdural silicone drainage catheters (12-French) performed under general anesthesia or conscious sedation. A single-drain configuration was employed when intraoperative cerebral expansion restricted the remaining subdural compartment. TDC under local anesthetic without irrigation was selectively utilized for medically high-risk patients and consisted of a 3.5 or 4.5 mm craniostomy with Trendelenburg position and Valsalva maneuver aiding in the evacuation of the hematoma. Middle meningeal artery embolization was not performed in any included patient. The designation of medically high-risk was based on the clinical judgment of the treating surgeon, integrating patient age, comorbidity burden, and the anticipated risk of anesthesia-related complications such as postoperative delirium or the need for prolonged ICU observation. No formal frailty scoring instrument was applied systematically, and no predefined objective criteria governed patient selection. This pragmatic approach reflects real-world practice but limits the reproducibility of the patient selection process.

Postoperative imaging during the initial hospitalization was routinely obtained following TDC and selectively after BHC procedures when new neurological changes emerged, preexisting deficits persisted, or inadequate decompression was clinically suspected. Substantial remaining hematoma with ongoing mass effect prompted repeat surgery before hospital discharge.

Patients were discharged following complete symptomatic improvement or when postoperative imaging confirmed the resolution of compressive effects. Outpatient surveillance CT imaging was scheduled 21 to 28 days after surgery, with a subsequent 4-week interval imaging until complete radiological clearance.

### 2.5. Primary Endpoint

The primary study endpoint was postoperative recurrence necessitating surgical reintervention. Recurrence was operationalized as volumetric expansion of residual or newly formed subdural fluid associated with imaging evidence of mass effect and/or development of new or progressive neurological symptoms requiring repeat operative drainage. Reoperations were performed via BHC with subdural drain insertion.

### 2.6. Statistical Methodology

#### 2.6.1. Descriptive and Univariate Analysis

All analyses were conducted using R version 4.5.2 (R Foundation for Statistical Computing, Vienna, Austria) through RStudio (version 2025.09.2+418, Posit Software, PBC, Boston, MA, USA). Core analytical packages included tidyverse, with MatchIt employed for propensity score methodology and cobalt utilized for balance diagnostics.

Continuous data were expressed as means with standard deviations (SD) when normally distributed or as medians with interquartile ranges (IQR, Q1 to Q3) when non-normally distributed. Distributional assumptions were verified through histogram visualization and Shapiro–Wilk testing where applicable. Categorical data were presented as counts and percentages.

Patient demographics, radiological characteristics, and surgical parameters were compared between recurrence and non-recurrence groups using conventional bivariate methods. Student’s *t*-tests were applied for normally distributed continuous data, Mann–Whitney U tests for non-normally distributed continuous data, and chi-square tests (or Fisher’s exact test when expected frequencies were <5) for categorical data.

#### 2.6.2. Multivariable Logistic Regression

For patients discharged following treatment solely by TDC, multivariable binary logistic regression was performed to identify the independent predictors of late recurrence. The dependent variable was recurrence requiring reoperation after initial discharge. Candidate predictors included GCS, hematoma architecture (organized type [14]), preoperative hematoma volume, and use of antithrombotic medication. These variables were selected on the basis of a priori clinical reasoning supported by the existing literature: GCS as a marker of neurological severity at presentation; hematoma architecture based on its established association with recurrence after surgical treatment [14]; hematoma volume as a consistently reported predictor in prior studies [6,7,8]; and antithrombotic therapy, given its recognized association with bleeding risk and hematoma membrane activity. Variable selection was not data-driven from the univariate analysis in order to minimize overfitting in a small sample. The model was constructed using complete case analysis, excluding patients with missing covariate data. Model discrimination was quantified using the area under the receiver operating characteristic curve (AUC), and calibration was assessed using the Hosmer–Lemeshow goodness-of-fit test. Variance inflation factors (VIF) were calculated to detect multicollinearity, with values exceeding 2.5 indicating problematic correlation. Results were reported as adjusted odds ratios (OR) with 95% confidence intervals (CI).

#### 2.6.3. Propensity Score Matching Analysis

Propensity score matching (PSM) was implemented to compare outcomes between TDC and BHC. The propensity score, defined as the conditional probability of receiving TDC given the observed baseline characteristics, was estimated via logistic regression.

Covariates included in the propensity model comprised demographic variables (age, sex), cardiovascular risk factors (hypertension, coronary artery disease, diabetes mellitus type 2), antithrombotic medications (antiplatelet therapy, oral anticoagulation), radiological hematoma features (volume, laterality, architecture classification, midline shift), and clinical severity indicators (GCS).

Matching was performed using one-to-one nearest-neighbor pairing without replacement, applying a caliper of 0.2 standard deviations of the logit propensity score to optimize match quality while preserving sample size. Covariate balance achievement was evaluated using standardized mean differences (SMD), with absolute SMD values below 0.1 indicating satisfactory balance.

Within the matched cohort, recurrence rates were compared using Fisher’s exact test. Additionally, conditional logistic regression stratified on matched pairs was performed to account for the paired structure of the matched data. Findings from the matched analysis were compared to the primary logistic regression results to assess consistency of treatment effects across analytical approaches.

Statistical significance was established at a two-sided alpha level of 0.05 for all hypothesis tests.

## 3. Results

### 3.1. Patient Cohort and Baseline Characteristics

**Figure 1:**
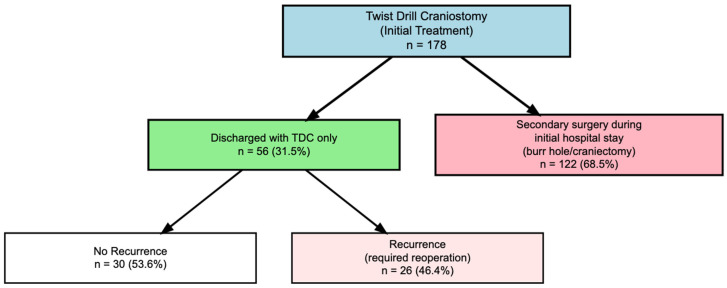
Patient flow diagram for twist drill craniostomy cohort. Of 178 patients initially treated with twist drill craniostomy (TDC), 56 (31.5%) were discharged without additional surgery during the index hospitalization, while 122 (68.5%) required conversion to burr hole craniotomy or craniectomy. Among patients discharged with TDC only, 30 (53.6%) remained recurrence-free, and 26 (46.4%) developed late recurrence requiring reoperation.

Between January 2015 and December 2023, 178 patients underwent TDC as initial treatment for cSDH at our institution. During the index hospitalization, 122 patients (68.5%) required conversion to BHC due to persistent symptoms or residual hematoma with mass effect, while 56 patients (31.5%) were successfully discharged following TDC alone (Figure 1). The analytical cohort for TDC-only outcomes comprised these 56 patients, with a mean age of 77.6 ± 9.7 years and included 19 females (33.9%) and 36 males (65.3%). Overall, 26 patients (46.4%) experienced late recurrence requiring surgical reintervention after hospital discharge. The combined early and late recurrence rate of TDC requiring BHC was 83.1%.

Baseline demographic characteristics, clinical presentation, hematoma imaging features, and comorbidity profiles for patients discharged following TDC only, stratified by recurrence status, are presented in Table 1. Patients who developed recurrence did not differ significantly from non-recurrent patients in age (75.9 ± 8.9 years vs. 79.1 ± 10.1 years, *p* = 0.219) or sex distribution (female: 34.6% vs. 34.5%, *p* = 1.000). Cardiovascular comorbidities showed similar prevalence between groups, including hypertension (57.7% vs. 62.1%, *p* = 0.956), coronary artery disease (30.8% vs. 37.9%, *p* = 0.784), and type 2 diabetes (11.5% vs. 10.3%, *p* = 1.000). The laboratory parameters, including activated partial thromboplastin time, international normalized ratio, and platelet count, did not differ between groups.

**Figure 2:**
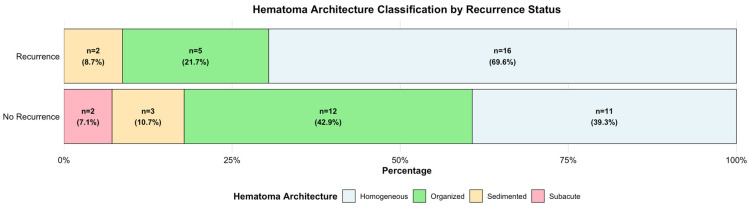
Distribution of hematoma architecture classification by recurrence status in patients discharged with twist drill craniostomy only. Horizontal stacked bar chart showing the distribution of the four collapsed hematoma architecture categories in patients without recurrence (*n* = 28) and with recurrence (*n* = 23). Organized hematomas (laminar, bridging, trabecular) were more prevalent in the no recurrence group (42.9% vs. 21.7%), while homogeneous hematomas (hypodense, isodense, hyperdense) were more common in the recurrence group (69.6% vs. 39.3%).

Regarding hematoma imaging characteristics, the median preoperative volume was comparable between groups (177.0 mL vs. 158.0 mL, *p* = 0.335). Hematoma laterality and midline shift presence showed no association with recurrence. Hematoma architecture demonstrated distinct distribution patterns between groups, where homogeneous hematomas (hypodense, isodense, hyperdense) comprised 69.6% of recurrent cases compared to 39.3% of non-recurrent cases (Figure 2).

### 3.2. Multivariable Logistic Regression Analysis

**Figure 3:**
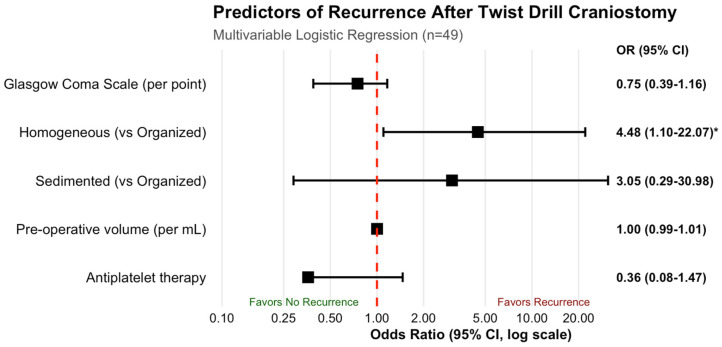
Multivariable logistic regression analysis of predictors of recurrence in patients discharged with twist drill craniostomy only. Forest plot showing adjusted odds ratios (OR) and 95% confidence intervals (CI) for predictors of recurrence after twist drill craniostomy (*n* = 49). Homogeneous hematomas (hypodense, isodense, hyperdense) had significantly higher odds of recurrence compared to organized hematomas (laminar, bridging, trabecular, subacute) as the reference category (OR 4.48, 95% CI 1.10–22.07, *p* = 0.037). Sedimented hematomas, Glasgow Coma Scale score, pre-operative hematoma volume, and antiplatelet therapy were not significantly associated with recurrence. The model showed acceptable discrimination (AUC 0.757) and good calibration (Hosmer–Lemeshow *p* = 0.925). * *p* < 0.05.

The independent predictors of recurrence following TDC were evaluated through multivariable binary logistic regression among the 49 patients with complete covariate data (Figure 3). Given the small number of subacute hematomas (*n* = 2, both without recurrence) causing estimation instability, the hematoma architecture variable was collapsed into three categories: organized (including subacute), homogeneous, and sedimented, with organized hematomas serving as the reference category.

In the multivariable model, homogeneous hematoma architecture emerged as a significant independent predictor of recurrence (adjusted OR 4.48, 95% CI 1.10–22.07, *p* = 0.037). Patients with homogeneous hematomas demonstrated 4.5-fold higher odds of recurrence compared to those with organized hematomas. Sedimented hematomas showed non-significantly elevated odds (adjusted OR 3.05, 95% CI 0.29–30.98, *p* = 0.431).

The Glasgow Coma Scale score was not significantly associated with recurrence (adjusted OR 0.75 per point, 95% CI 0.39–1.16, *p* = 0.296). Preoperative hematoma volume showed no association with recurrence (adjusted OR 1.00 per mL, 95% CI 0.99–1.01, *p* = 0.693). Antiplatelet therapy was not significantly associated with recurrence (adjusted OR 0.36, 95% CI 0.08–1.47, *p* = 0.137).

The model demonstrated acceptable discrimination (area under the receiver operating characteristic curve 0.757, 95% CI 0.620–0.893) and good calibration (Hosmer–Lemeshow test χ^2^ = 3.14, *p* = 0.925). Variance inflation factors for all covariates were below 2.0, indicating the absence of problematic multicollinearity.

### 3.3. Propensity Score Matching Analysis

To compare outcomes between TDC and BHC while controlling for selection bias, propensity score matching was performed. After excluding patients with missing data in matching variables, 387 patients remained eligible: 50 TDC patients (discharged with TDC only) and 337 burr hole craniotomy patients. Baseline assessment revealed substantial imbalances between treatment groups, with SMDs exceeding 0.1 for several variables, including hematoma architecture (SMD −0.50), age (SMD 0.41), and preoperative volume (SMD 0.37), indicating meaningful baseline differences requiring adjustment (Supplemental Figure S1).

One-to-one nearest neighbor propensity score matching with a caliper of 0.2 standard deviations successfully matched 48 TDC patients with 48 burr hole patients, yielding a matched cohort of 96 patients (Table 2). Only 2 TDC patients (4%) remained unmatched due to a lack of sufficiently similar controls. Post-matching balance assessment demonstrated excellent covariate balance, with all standardized mean differences below 0.2 (Supplemental Figure S1). Most variables achieved standardized mean differences below 0.1, with hypertension (SMD 0.13) and GCS (SMD −0.19) showing slightly elevated, but acceptable, values.

Baseline characteristics in the matched cohort confirmed successful balancing (Table 2). The mean age was 78.2 ± 9.2 years in the TDC group versus 77.9 ± 11.1 years in the burr hole group. Comorbidity prevalence, anticoagulation use, and hematoma imaging parameters demonstrated comparable distributions between matched groups. Notably, the organized hematoma frequencies were identical (39.6% in both groups), confirming successful matching on this critical prognostic variable.

**Figure 4:**
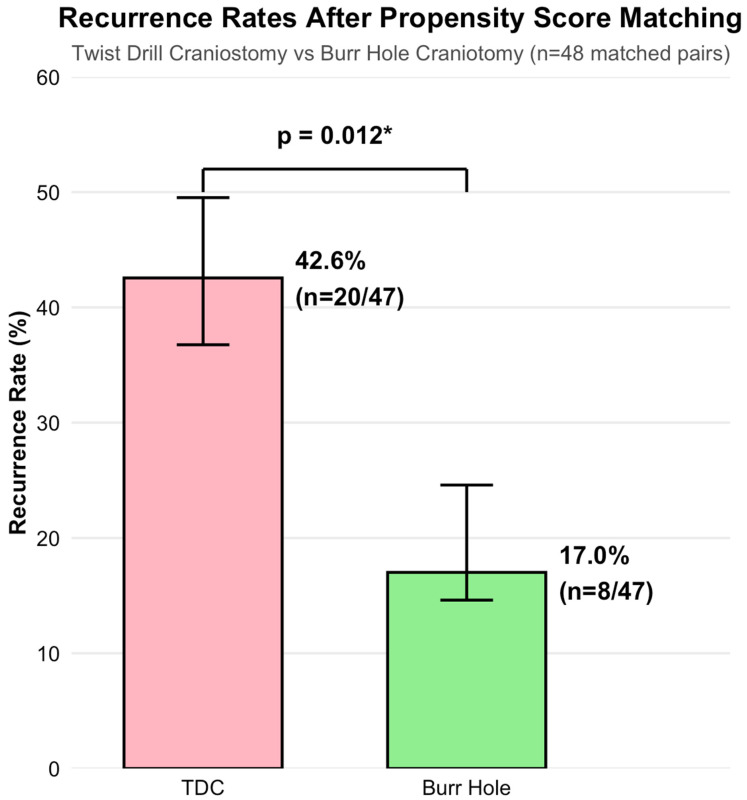
Recurrence rates in matched cohort: twist drill craniostomy versus burr hole craniotomy. Comparison of recurrence rates in 48 propensity score matched pairs. Twist drill craniostomy (TDC) had significantly higher recurrence than burr hole craniotomy (42.6% vs. 17.0%, *p* = 0.012). Error bars represent 95% confidence intervals. * *p*-value < 0.05.

In the matched cohort, the recurrence rates differed markedly between treatment approaches (Figure 4). TDC patients experienced recurrence in 42.6% of cases (20/47), compared to 17.0% (8/47) among the burr hole craniotomy patients (*p* = 0.012, Fisher’s exact test). This corresponded to an unadjusted OR of 3.56 (95% CI 1.28–10.80), favoring burr hole craniotomy.

Conditional logistic regression accounting for the matched-pair structure confirmed this association (adjusted OR 3.20, 95% CI 1.17–8.73, *p* = 0.023). The concordance statistic of 0.762 indicated good discriminative ability.

## 4. Discussion

In this retrospective cohort study of patients undergoing twist drill craniostomy for the treatment of chronic subdural hematoma, the procedure was associated with a high rate of hematoma recurrence requiring reoperation. Here, homogeneous hematoma architecture emerged as the only independent predictor of recurrence. When compared to burr hole craniotomy patients using propensity score matching to adjust for baseline differences in demographics, comorbidities, and hematoma characteristics, twist drill craniostomy was associated with higher recurrence rates.

Recent randomized controlled trials have established comprehensive, evidence-based principles for the surgical management of cSDH. The multicenter COMPACT trial compared three surgical techniques in 245 patients, demonstrating that burr hole craniostomy yielded, numerically, the lowest reoperation rate (7.6%) compared to minicraniotomy (13.1%) and twist drill craniostomy (19.5%) [18]. Twist drill craniostomy offered significantly shorter operative time (mean 19.9 min vs. 52.9 min for burr hole), with comparable 6-month functional outcomes. The FINISH trial evaluated subdural irrigation in 589 patients, demonstrating a 6% higher reoperation rate when irrigation was omitted, favoring irrigation use despite minimal impact on operative time [3]. In a Swedish trial further refining the irrigation technique in 541 patients, irrigation fluid at body temperature (37 °C) significantly reduced recurrence compared to room temperature (22 °C) without differences in mortality or complications [4]. The cSDH-Drain trial demonstrated that subperiosteal drains achieved lower recurrence rates (8.3% versus 12.0%) with significantly fewer surgical infections and drain misplacements compared to subdural drains, though formal non-inferiority criteria were narrowly not met [19]. A Danish trial evaluated drainage duration in 420 patients, finding no significant difference in recurrence or mortality, while 24 h drainage resulted in significantly shorter neurosurgical length of stay (2.0 versus 2.8 days) [5]. Finally, the field of primary or adjunct middle meningeal artery embolization is rapidly expanding. The EMBOLISE trial evaluated adjunctive middle meningeal artery embolization in 400 patients, demonstrating significantly reduced reoperation rates with embolization plus surgery compared to surgery alone (4.1% versus 11.3%), though serious adverse events related to embolization occurred in 2.0% of patients, including disabling stroke in two patients [20]. Collectively, these trials establish BHC with body temperature irrigation, 24 h postoperative drainage, and consideration of adjunctive middle meningeal artery embolization (in selected patients) as evidence-based surgical practice for cSDH.

While RCTs provide the highest level of evidence, these guidelines necessarily represent a ‘one-size-fits-all’ approach derived from populations meeting strict inclusion criteria. Elderly patients with cSDH, however, represent a heterogeneous population with varying degrees of frailty, medical comorbidity, and physiologic reserve. The exclusion of high-risk patients from major trials, such as those unable to provide informed consent, those with significant baseline functional impairment, or those with multiple comorbidities, creates a potential evidence gap for precisely the patients who might benefit most from less invasive surgical approaches. TDC, with its shorter operative time, reduced anesthetic requirements, and potential for performance under local anesthesia, theoretically offers advantages for this medically fragile subpopulation. The critical question, therefore, is whether these theoretical benefits translate into acceptable clinical outcomes or whether the increased risk of recurrence outweighs the procedural advantages.

Multiple systematic reviews and meta-analyses have examined the comparative efficacy of TDC versus BHC. Yagnik et al. conducted a comprehensive meta-analysis of 16 studies (1235 patients) comparing both techniques, finding no significant differences in mortality, recurrence, cure rates, or complications between the two approaches [21]. However, TDC was associated with higher overall reoperation rates. Critically, subgroup analysis revealed that this disadvantage was eliminated when TDC was performed with negative suction drainage. This finding aligns with Wei et al.’s meta-analysis of 2536 patients examining TDC with hollow screws, which demonstrated that, while overall success and recurrence rates were similar between TDC and BHC, the hollow screw subgroup specifically achieved 50% lower recurrence rates (*p* = 0.002) compared to BHC [22].

Single-center experiences support these meta-analytic findings while highlighting important technical considerations. A historical retrospective analysis of 233 patients treated at our center with TDC demonstrated a 67% overall success rate, with smaller hematoma volume emerging as the primary predictor of treatment success [23].

The collective observational evidence suggests that TDC efficacy is influenced by not only patient-related but also technical factors, particularly the drainage system employed. The superiority of negative suction drainage over passive systems appears to address the fundamental challenge of adequate hematoma evacuation through smaller bone openings (3–5.8 mm for TDC vs. 10–25 mm for BHC). Meanwhile, an organized hematoma composition does not appear to be a contraindication.

Our findings both complement and extend the existing literature on TDC, while revealing important discrepancies that warrant careful consideration. The high recurrence rate observed in our TDC cohort aligns with the COMPACT trial results and meta-analytic data demonstrating increased reoperation risk with TDC. However, our study identifies a critical gap between the theoretical promise of TDC and its real-world implementation.

Contrary to what might be expected, homogeneous hematomas were associated with the highest recurrence rates in our cohort. The fluid nature of homogeneous collections facilitates initial passive drainage through the small twist drill opening, yet does not appear to confer protection against reaccumulation. If TDC is considered in this context, patients and families should be counseled that the procedure may provide rapid relief of mass effect but carries a substantially elevated risk of recurrence, and that reoperation is likely.

### Limitations

The retrospective single-center design of this study inherently introduces risks of selection bias and unmeasured confounding. Although PSM was performed to reduce baseline imbalances between TDC and BHC cohorts, residual confounding from factors not captured in the dataset, such as frailty status, functional baseline, surgeon-specific techniques, or subtle radiographic features, cannot be excluded.

Patients who required conversion to BHC during the index hospitalization were classified as early treatment failures and excluded from the recurrence analysis, rather than being counted as recurrences. This reflects a conceptual distinction between early procedural failure, whereby the initial TDC did not achieve sufficient evacuation to permit discharge, and late biological recurrence in patients who were successfully discharged following TDC. Pooling them into a single recurrence endpoint would risk obscuring the biological predictors of late failure that were the primary focus of this investigation. Nonetheless, this definition restricts the multivariable analysis to the 56 patients (31.5%) who achieved sufficient initial evacuation, a selected subgroup with, by definition, a favorable early response to TDC. This introduces selection bias into the regression analysis, and the identified predictors should be interpreted as predictors of late recurrence within this subgroup rather than as predictors of overall TDC outcome. The combined early and late retreatment rate of 83.1% reflects the full burden of TDC failure across both time horizons.

The study population undergoing definitive TDC was relatively small. Of the 178 patients initially treated with TDC, only 56 were discharged without having received additional surgery, and complete follow-up data were available for 55 patients. This limited sample size reduces statistical power, widens confidence intervals, and restricts the number of predictors that can be reliably assessed in multivariable modeling. The multivariable logistic regression was conducted on 49 complete cases with 26 recurrence events, yielding an events-per-variable ratio of approximately 6 to 7. The wide confidence intervals observed, most notably for homogeneous hematoma architecture (OR 4.48, 95% CI 1.10 to 22.07) and sedimented hematoma (OR 3.05, 95% CI 0.29 to 30.98), reflect this limitation. The regression findings should, therefore, be interpreted as hypothesis-generating and associative rather than definitive, and replication in larger prospective cohorts is warranted.

Finally, this study focused on recurrence requiring reoperation as the primary endpoint. Functional outcomes, quality of life, delirium rates, anesthetic complications, and cost-effectiveness were not systematically captured, limiting conclusions regarding the broader comparative value of TDC in frail or high-risk populations. This is a relevant constraint because the principal rationale for TDC in elderly patients relates not only to recurrence but also to avoidance of general anesthesia, reduction in perioperative delirium risk, shorter procedural duration, and lower physiological burden. These benefits are difficult to assess retrospectively, and they are further complicated by the fact that a patient who avoids general anesthesia with TDC but subsequently requires BHC weeks later has experienced both the acute procedural risks of TDC and the delayed anesthetic burden of BHC. A comprehensive evaluation of TDC as a treatment strategy for frail patients would require prospective capture of functional status, delirium incidence, length of stay, and patient-reported outcomes, alongside formal frailty assessment.

## 5. Conclusions

In conclusion, TDC can provide definitive treatment in a small subset of patients with cSDH. However, in our real-world institutional experience, recurrence after TDC was frequent, and homogeneous hematoma architecture emerged as the strongest predictor of treatment failure. Even after adjustment for baseline differences using propensity score matching, TDC was associated with significantly higher recurrence rates compared with BHC. These findings reinforce burr hole evacuation with irrigation and drainage as the evidence-based standard of care, while suggesting that TDC should be reserved for carefully selected patients. However, the criteria by which this selection should take place remain elusive.

## Figures and Tables

**Table 1:** Baseline characteristics stratified by recurrence Status.

Variable	No Recurrence (*n* = 30)	Recurrence (*n* = 26)	*p* Value
**Demographics**			
Age (years)	79.1 ± 10.1	75.9 ± 8.9	0.219
Sex—Female	10 (34.5)	9 (34.6)	1.000
**Comorbidity**			
Hypertension	18 (62.1)	15 (57.7)	0.956
Coronary artery disease	11 (37.9)	8 (30.8)	0.784
Diabetes mellitus type 2	3 (10.3)	3 (11.5)	1.000
Peripheral artery disease	3 (10.3)	7 (26.9)	0.164
Antiplatelet therapy	13 (44.8)	6 (23.1)	0.159
Oral anticoagulation	2 (6.9)	2 (7.7)	1.000
aPTT (s)	27.8 ± 3.4	28.8 ± 4.2	0.354
INR	1.0 (0.9 to 1.1)	1.0 (1.0 to 1.1)	0.275
Platelet count (×10^9^/L)	230.7 ± 71.4	248.6 ± 60.6	0.328
**Hematoma characteristics**			
Hematoma side—Left	12 (41.4)	8 (36.4)	1.000
Hematoma side—Bilateral	11 (37.9)	9 (40.9)	1.000
Maximum width (mm)	24.0 (20.0 to 28.0)	24.5 (21.0 to 28.2)	0.320
Pre-operative volume (mL)	158.0 (123.0 to 189.0)	177.0 (124.8 to 238.2)	0.335
Midline shift present	26 (89.7)	21 (80.8)	0.455
Midline shift (mm)	9.0 ± 2.6	9.3 ± 3.8	0.709
**Hematoma architecture**			
Homogeneous	11 (39.3)	16 (69.6)	
Organized	12 (42.9)	5 (21.7)	
Sedimented	4 (14.3)	2 (8.7)	
Subacute	1 (3.6)	0 (0.0)	
**Clinical presentation**			
GCS	15.0 (15.0 to 15.0)	15.0 (14.0 to 15.0)	0.004
Emergency surgery	3 (10.3)	3 (11.5)	1.000
Pre-operative paresis	16 (57.1)	17 (68.0)	0.596
Pre-operative headache	12 (42.9)	13 (52.0)	0.697
Pre-operative aphasia	6 (21.4)	8 (32.0)	0.576

Data are presented as mean ± standard deviation for normally distributed continuous variables, median (interquartile range) for non-normally distributed continuous variables, and number (percentage) for categorical variables. Extended hematoma type classification: homogeneous (hypodense, isodense, hyperdense), organized (laminar, bridging, trabecular), sedimented, and subacute. aPTT, activated partial thromboplastin time; GCS, Glasgow Coma Scale; INR, international normalized ratio; mm, millimeter; mL, milliliter.

**Table 2:** Baseline characteristics before and after propensity score matching.

Characteristic	TDC (*n* = 50)	BHC (*n* = 337)	TDC (*n* = 48)	BHC (*n* = 48)
	Before Matching	Before Matching	After Matching	After Matching
Sample size	50	337	48	48
**Demographics**				
Age (years), mean ± SD	78.3 ± 9.2	74.6 ± 12.5	78.2 ± 9.2	77.9 ± 11.1
Female sex, *n* (%)	20 (40.0)	116 (34.4)	18 (37.5)	20 (41.7)
**Comorbidities**				
Hypertension	30 (60.0)	216 (64.1)	29 (60.4)	23 (47.9)
Coronary artery disease	17 (34.0)	102 (30.3)	15 (31.2)	16 (33.3)
Diabetes mellitus	4 (8.0)	62 (18.4)	4 (8.3)	6 (12.5)
Antiplatelet therapy	16 (32.0)	109 (32.3)	16 (33.3)	12 (25.0)
Oral anticoagulation	4 (8.0)	52 (15.4)	4 (8.3)	7 (14.6)
**Hematoma characteristics**				
Pre-operative volume (mL), median (IQR)	158 (124 to 200)	130 (92 to 180)	158 (122 to 197)	165 (114 to 203)
Organized hematoma, *n* (%)	19 (38.0)	210 (62.3)	19 (39.6)	19 (39.6)
Midline shift present, *n* (%)	43 (86.0)	258 (76.6)	41 (85.4)	42 (87.5)
**Clinical presentation**				
Glasgow Coma Scale, median (IQR)	15 (14 to 15)	15 (14 to 15)	15 (14 to 15)	15 (15 to 15)
**Outcome**				
Recurrence, *n* (%)	21 (42.9)	46 (13.9)	20 (42.6)	8 (17.0)

Comparison of patient demographics, comorbidities, hematoma characteristics, and clinical presentation between twist drill craniostomy (TDC) and burr hole craniotomy patients before and after 1:1 propensity score matching. Continuous variables are presented as mean ± standard deviation or median (interquartile range). Categorical variables are presented as *n* (%). After matching, 48 pairs were successfully created with good balance achieved across all baseline covariates (standardized mean differences < 0.2). BHC, burr hole craniotomy; IQR, interquartile range; mL, milliliter; SD, standard deviation; TDC, twist drill craniostomy.

## Data Availability

The raw data of this analysis can be made available by the authors to any qualified researcher upon reasonable request.

## Supplementary Materials

The following supporting information can be downloaded at https://www.mdpi.com/article/10.3390/brainsci16050516/s1: Figure S1: Covariate balance before and after propensity score matching.

## Abbreviations

AUC	area under the curve
BHC	burr hole craniotomy
CI	confidence interval
cSDH	chronic subdural hematoma
CT	computed tomography
GCS	Glasgow Coma Scale
IQR	interquartile range
mL	milliliter
mm	millimeter
OR	odds ratio
PSM	propensity score matching
SD	standard deviation
SMD	standardized mean difference
TDC	twist drill craniostomy
VIF	variance inflation factor
