# Supplementary material for: Is There Still a Role for Twist Drill Craniostomy in Contemporary Management of Chronic Subdural Hematoma?
Source: Brain Sci. 2026 May 12;16(5):516. doi: 10.3390/brainsci16050516 (PMC13204861; doi:10.3390/brainsci16050516)
Supplement: Supplementary file 1 [file brainsci-16-00516-s001.zip › brainsci-4285153-supplementary.pdf]

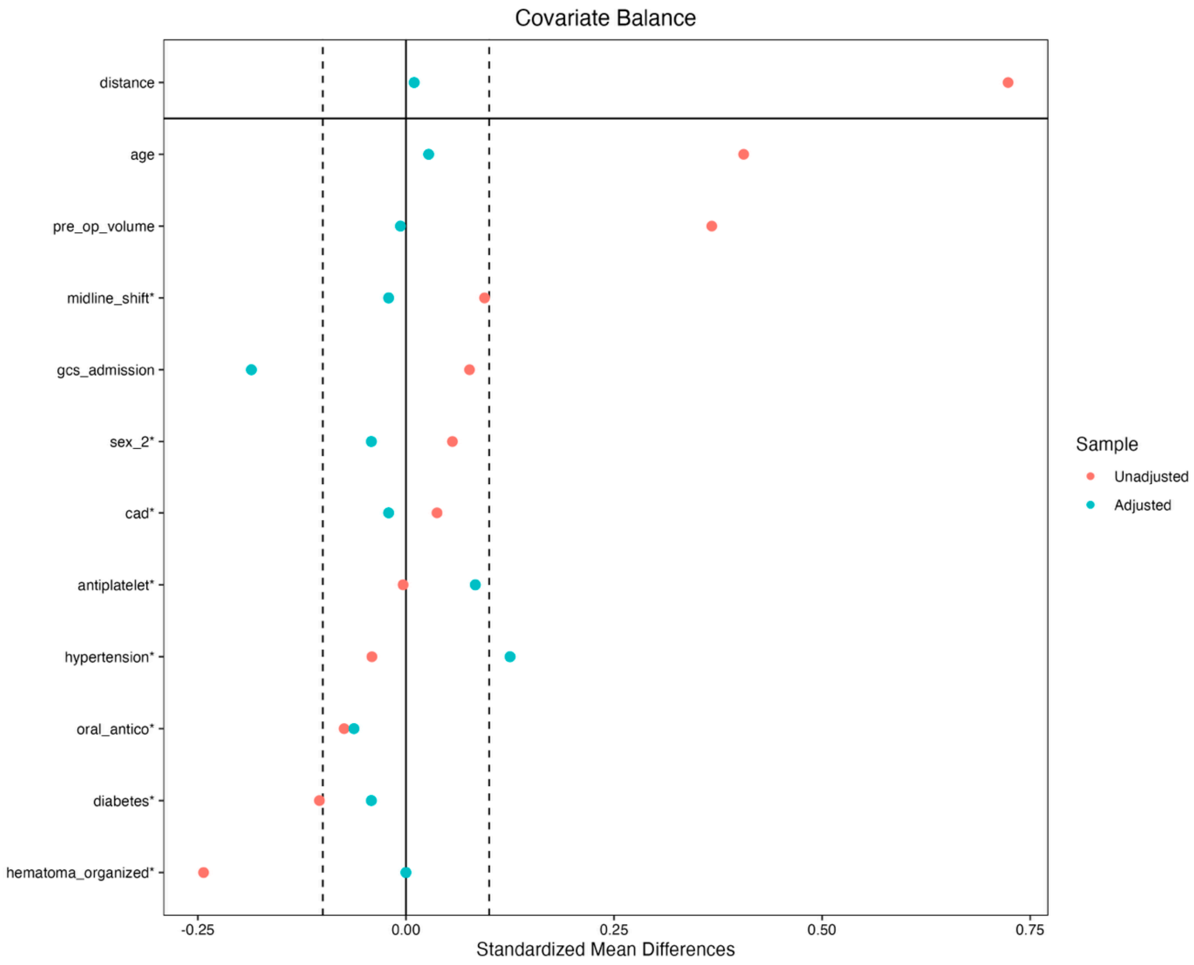

Supplemental Figure S1. Covariate balance before and after propensity score matching. Love plot displaying standardized mean differences (SMD) for baseline covariates before (unadjusted, red) and after (adjusted, blue) 1:1 propensity score matching between twist drill craniostomy (n=48) and burr hole craniotomy (n=48) patients. The vertical dashed lines represent the threshold for acceptable balance (SMD = ±0.1). After matching, most covariates achieved good balance with SMD < 0.1, indicating successful matching. Variables are ordered by unadjusted SMD magnitude. The propensity score (distance) and hematoma architecture (hematoma_organized) showed the largest improvement in balance after matching, with SMD reducing from 0.72 to 0.01 and -0.24 to 0.00, respectively. Two variables (hypertension and GCS admission) had SMD slightly exceeding 0.1 after matching but remained within acceptable limits for valid comparison. CAD, coronary artery disease; GCS, Glasgow Coma Scale.
